# Register-based and genetic studies of Prader-Willi syndrome show a high frequency of gonadal tumors and a possible mechanism for tumorigenesis through imprinting relaxation

**DOI:** 10.3389/fmed.2023.1172565

**Published:** 2023-07-28

**Authors:** Carolina Maya-González, Sandra Wessman, Kristina Lagerstedt-Robinson, Fulya Taylan, Bianca Tesi, Ekaterina Kuchinskaya, W. Glenn McCluggage, Anna Poluha, Stefan Holm, Ricard Nergårdh, Teresita Díaz De Ståhl, Charlotte Höybye, Giorgio Tettamanti, Angelica Maria Delgado-Vega, Anna Skarin Nordenvall, Ann Nordgren

**Affiliations:** ^1^Department of Molecular Medicine and Surgery, Center for Molecular Medicine, Karolinska Institutet, Stockholm, Sweden; ^2^Department of Oncology-Pathology, Karolinska Institutet, Stockholm, Sweden; ^3^Department of Pathology and Cancer Diagnostics, Karolinska University Hospital, Stockholm, Sweden; ^4^Department of Clinical Genetics, Karolinska University Hospital, Stockholm, Sweden; ^5^Department of Medicine Huddinge, Karolinska Institutet, Stockholm, Sweden; ^6^Department of Clinical Experimental Medicine, Linköping University, Linköping, Sweden; ^7^Department of Pathology, Belfast Health and Social Care Trust, Belfast, United Kingdom; ^8^Clinical Genetics, Uppsala University Hospital, Uppsala, Sweden; ^9^Department of Immunology, Genetics and Pathology, Faculty of Medicine, Uppsala University, Uppsala, Sweden; ^10^Department of Women's and Children's Health, Karolinska Institutet, Stockholm, Sweden; ^11^Department of Endocrinology, Karolinska University Hospital, Stockholm, Sweden; ^12^Unit of Epidemiology, Institute of Environmental Medicine, Karolinska Institutet, Stockholm, Sweden; ^13^Department of Radiology, Karolinska University Hospital, Stockholm, Sweden; ^14^Department of Laboratory Medicine, Institute of Biomedicine, University of Gothenburg, Gothenburg, Sweden; ^15^Department of Clinical Genetics and Genomics, Sahlgrenska University Hospital, Gothenburg, Sweden

**Keywords:** Prader-Willi syndrome, ovarian tumor, germ-cell tumor, cancer predisposition, loss-of-imprinting, imprinting relaxation

## Abstract

Prader-Willi syndrome (PWS) is a rare disease caused by a lack of expression of inherited imprinted genes in the paternally derived Prader-Willi critical region on chromosome 15q11.2-q13. It is characterized by poor feeding and hypotonia in infancy, intellectual disability, behavioral abnormalities, dysmorphic features, short stature, obesity, and hypogonadism. PWS is not a known cancer predisposition syndrome, but previous investigations regarding the prevalence of cancer in these patients suggest an increased risk of developing specific cancer types such as myeloid leukemia and testicular cancer. We present the results from a Swedish national population-based cohort study of 360 individuals with PWS and 18,000 matched comparisons. The overall frequency of cancer was not increased in our PWS cohort, but we found a high frequency of pediatric cancers. We also performed whole-genome sequencing of blood- and tumor-derived DNAs from a unilateral dysgerminoma in a 13-year-old girl with PWS who also developed bilateral ovarian sex cord tumors with annular tubules. In germline analysis, there were no additional findings apart from the 15q11.2-q13 deletion of the paternal allele, while a pathogenic activating *KIT* mutation was identified in the tumor. Additionally, methylation-specific multiplex ligation-dependent probe amplification revealed reduced methylation at the PWS locus in the dysgerminoma but not in the blood. In conclusion, our register-based study suggests an increased risk of cancer at a young age, especially testicular and ovarian tumors. We found no evidence of a general increase in cancer risk in patients with PWS. However, given our limited observational time, further studies with longer follow-up times are needed to clarify the lifetime cancer risk in PWS. We have also described the second case of locus-specific loss-of-imprinting in a germ cell tumor in PWS, suggesting a possible mechanism of carcinogenesis.

## 1. Introduction

Prader-Willi syndrome (PWS, OMIM #176270) is a multisystemic genetic disorder caused by imprinting defects in paternally expressed genes in the 15q11.2-q13 region (1). In the neonatal period, patients present with poor sucking, failure to thrive, and severe hypotonia. Subsequently, they develop hyperphagia which, in the natural course of disease, leads to obesity. Additional symptoms include craniofacial dysmorphisms, short stature, small hands and feet, hypogonadism with cryptorchidism in male sex, intellectual disability of variable degree, and behavioral changes (1, 2). The genetic causes of PWS include paternal 15q11.2-q13 deletions, maternal uniparental disomy (UPD) 15, and imprinting defects (3), all leading to loss of expression of the paternal-only expressed genes *MKRN3, MAGEL2, NECDIN, SNURF, SNRPN* and seven non-coding RNAs (1).

Dysgerminomas are ovarian germ cell tumors that occur in female sex, primarily in teenagers and young women (4), and sometimes in patients with gonadal dysgenesis. Sex cord tumor with annular tubules (SCTATs) are rare ovarian tumors with low malignant potential. These tumors are strongly associated with Peutz-Jeghers syndrome, which is present in more than one-third of all cases (5). SCTATs have also been described in Turner syndrome, as well as in combination with other neoplasms, including dysgerminomas and gonadoblastomas (6). However, they have rarely been reported in children (7).

Genomic imprinting is an epigenetic mechanism that controls the monoallelic expression of specific genomic regions according to their parental origin and implies hemizygous inheritance of the imprinted genes (8). During gametogenesis, parent-specific imprinting is established by diverse mechanisms, including DNA methylation and chromatin remodeling by histone modification (9). Parent-of-origin-imprinted marks are maintained throughout the entire life of an organism, though erased in the gonads, before new imprinting is set during gametogenesis according to the gender (10). In the recent decades, imprinted genomic regions have been mapped (11), highlighting their relevance in disease and cancer (12). In certain conditions, such as carcinogenesis, imprinting marks can be lost. This process is known as imprinting relaxation or loss-of-imprinting (LOI). During carcinogenesis, LOI is associated with the activation of silent oncogenes or the inactivation of normally expressed tumor suppressor genes (TSG) (13).

The risk of cancer in patients with PWS is poorly understood, and the condition is not considered a cancer predisposition syndrome. Nevertheless, previous studies have indicated an overrepresentation of malignancies in this patient group (14–16), and individual case reports of cancer in PWS exist (Supplementary Table 1). Specifically, two American studies on individuals with PWS, one including 531 male patients and another with 1,160 patients, reported an increased relative risk of testicular cancer of 13.5 (15) and a higher incidence of leukemia (16) in individuals with PWS, respectively. Finally, a study of the Finnish population with 56 individuals with PWS reported three cancer cases, including a patient with testicular cancer and one with leukemia, which was twice the expected number of diagnoses (14). Furthermore, the risk of cancer development in individuals with PWS was possibly underestimated due to their relatively short life expectancy and the multiple co-morbidities accompanying the disease (1, 17).

In this study, we aimed to investigate the prevalence of cancer in PWS by using nationwide register-based data with information regarding PWS and cancer diagnoses. Furthermore, we present the second case of locus-specific LOI in DNA from the dysgerminoma of a 13-year-old girl with PWS and multiple ovarian tumors, namely, bilateral SCTATs and a dysgerminoma.

## 2. Materials and methods

### 2.1. Register-based study

#### 2.1.1. National registers used

In Sweden, all permanent residents are given unique personal identity numbers (PIN). Upon ethical approval, the PIN enables linkage between different national registers where data on demographics and healthcare are collected continuously (18). In 1964, the Swedish National Board of Health and Welfare began collecting data on all inpatient visits at public Swedish hospitals in the Sweden National Patient Register (NPR) (19). This register has nationwide coverage from 1987 onwards, including outpatient visits since 2001. Since 1973, the Board also maintains a nationwide Medical Birth Registry (MBR) of all pregnancies resulting in childbirth, including information on congenital malformations and perinatal diagnoses (20).

The National Cancer Register (NCR) was founded in 1958 and covers all cancer cases in Sweden (21). It is mandatory for all Swedish caregivers to report new cancer cases to the NCR. The register contains information on the tumor site (according to the International Classification of Diseases ICD-7, ICD-O/2, or ICD-O/3), histology (according to WHO/HS/CANC/24.1, ICD-O/2, or ICD-O/3), and date of diagnosis. Additionally, the Karolinska University laboratory information system (LIS) holds a clinical biobank with data on a large cohort of patients affected by different rare diseases. The biobank includes information on disease-causing mutations and phenotypes in 16,502 cases.

Statistics Sweden holds information on the date of birth, death, and emigration of all citizens in the Total Population Register (22). Furthermore, it keeps registers including data on educational level [the Longitudinal Integration Database for Health Insurance and Labor Market Studies; 1990 and onwards (23)] and across multiple generations [the Multi Generation Register (24)], enabling linkage between index individuals and their biological parents.

#### 2.1.2. Exposure and outcome

All individuals diagnosed with PWS were identified from the MBR and NPR. Since only ICD-10 includes a specific code for PWS (Q87.1F), solely patients diagnosed with PWS between 1997 and 2017 were included. Individuals who had been diagnosed with chromosomal aberrations after the last date of diagnosis of PWS were excluded. Furthermore, patients with a genetically confirmed PWS identified through the Karolinska University Hospital LIS, though lacking a PWS diagnosis in the NPR, were added to the patient cohort. Each patient was matched with 50 unaffected individuals by year of birth, sex, and birth county. Data on cancer diagnosis, tumor site, histology, and age at diagnosis were collected from the NCR. In the case of multiple cancer diagnoses, only the first was used for further analyses. Data on demographics and parental level of education were collected from Statistics Sweden. In the restricted analyses, only individuals born in Sweden between 1961 and 2017 with at least two registered diagnoses of PWS in the NPR or genetically confirmed PWS were included.

#### 2.1.3. Relationship between PWS and cancer: statistics

The association between PWS and cancer was estimated using a Cox proportional hazard model with the attained age as the underlying time scale. The results were presented as hazard ratios (HR) with 95% confidence intervals (CI), crude and adjusted for birth year, sex, and parental educational level. Each individual was followed from birth to the outcome event, namely, emigration, death, or end of the study period (31 December 2017), whichever occurred first. As defined by the World Health Organization, pediatric and adult cancers were considered as any malignancy before and after the age of 20 years, respectively (25). The validity of the proportional hazards assumption was verified by Schoenfeld residuals. All analyses were performed with the Stata statistical software, version 14.

### 2.2. Tumor histological and immunohistochemical assessment

The tumors in the 13-year-old girl with PWS were diagnosed through routine clinical assessment and pathological workup. The large tumor of the right ovary was diagnosed using a fine needle aspiration and a core biopsy. The former material was evaluated using May-Grünwald-Giemsa staining, and the latter with hematoxylin—eosin staining. Further processing with immunocytochemical and immunohistochemical markers was performed using antibodies for OCT3/4, SALL4, CD117, CD30, and Glypican 3. The bilateral microscopic SCTATs were diagnosed on surgically resected bilateral oophorectomies performed after preoperative chemotherapy. Immunohistochemical staining with markers such as inhibin, calretinin, SF1, MelanA, SALL4, and Ki67 was performed. Furthermore, molecular analysis by Sanger sequencing was performed to rule out *FOXL2* C134W mutation.

### 2.3. Whole genome sequencing and bioinformatic analysis

Patient-matched genomic DNAs from fresh frozen tumors and peripheral blood were extracted according to standard procedures. Paired 2 × 151 bp whole genome sequencing (WGS) was performed on NovaSeq 6000 (Illumina) instruments starting with 1 μg DNA and using the TruSeq DNA PCR-Free library preparation. Extracted tumor and blood DNAs were sequenced at 90 × /30 × coverage, respectively, as described in Stranneheim et al. (26). The choice of coverage for WGS is based on data from previous studies (26–28). The WGS of germline DNA was performed to confirm PWS diagnosis and exclude disease-causing variants in childhood cancer predisposition genes. The Scout platform (Clinical Genomics) was used for the ranking, visualization, and filtering of variants. The filtering out criteria included a minor allele frequency of above 0.01 in the general population (29), annotations outside coding and splice regions, and predicted benign/likely benign polymorphisms. Candidate variants were manually explored in Scout (Clinical Genomics) and visualized using the Integrative Genomics Viewer (IGV) (30).

The dysgerminoma sequencing data were analyzed using Balsamic (the Bioinformatic Analysis pipeLine for SomAtic MutatIons in Cancer, Clinical Genomics; https://github.com/Clinical-Genomics/BALSAMIC), and variants were filtered and visualized in Scout, as described in Stranneheim et al. (26). Tumor and copy-number changes were detected with the Control Freec software, v11.6.

### 2.4. Methylation-specific multiplex ligation-dependent probe amplification (MS-MLPA)

The imprinting status at the PWS locus was evaluated by MS-MLPA. DNAs derived from tumor and blood samples were analyzed using SALSA MLPA Probemix Prader-Willi/Angelman panel (MCR Holland, ME028), according to the manufacturer's instructions. As a control-imprinted region, the SALSA MLPA Probemix for the Beckwith-Wiedemann/Silver Russell Syndrome region (MCR Holland, ME030) was evaluated. The results were analyzed with the GeneMarker V2.7.0 software (SoftGenetics). In this software, probes with a peak ratio of 0.75–1.30 are generally considered to be within the normal interval.

## 3. Results

### 3.1. Register-based study

A total of 360 patients with PWS were identified through the MBR, NPR, or Karolinska University LIS, whereof 261 fulfilled the criteria for inclusion in the restricted cohort. The mean age at the end of follow-up was 24 years in PWS and 26 years in age-matched comparisons. The overall frequency of cancer was similar between patients and comparisons (Tables 1, 2), and there was no increased risk of cancer at any age in either the full or restricted cohort in time-to-event analyses (HR 1.07, 95% CI 0.6–1.9) (Table 3). However, pediatric cancer was more prevalent among PWS patients in the full cohort (3 cases) (Tables 1, 2). Among the individuals developing cancer, the occurrence of malignancies before the age of 20 years was observed in 3/12 (25%) of patients with PWS, as compared to 48/551 (8.7%) in the comparison group. However, the size of the affected cohort was too small to perform any further statistical analyses. Therefore, studies on larger cohorts will be needed to validate these results.

**Table 1:** Baseline characteristics of the full cohort.

	**PWS**		**Matched controls**	
	**N** = **360**	**%**	**N** = **17,963**	**%**
**Sex**				
Boys/Men	190	52.78%	9,463	52.68%
Girls/Women	170	47.22%	8,500	47.32%
**Birth region**				
Sweden	345	95.83%	17,250	96.03%
Other	15	4.17%	713	3.97%
**Cancer**				
Cancer at any age	12	3.33%	551	3.07%
Mean age at first cancer diagnosis (SD)	35.8 (±22.9)		38.7 (±16.7)	
Pediatric cancer (< 20 years)	3	0.83%	48	0.27%
**Emigration**				
Emigrated	9	3%	1,347	7%
Mean age at emigration (SD)	8.9 (±6.5)		17.0 (±13.3)	
**Death**				
Dead	43	12%	382	2%
Mean age at death (SD)	41.3 (±19.0)		37.3 (±24.3)	
31 December 2017	310	86%	16,247	90%
Mean age at the end of follow-up (SD)	24.4 (±16.1)		26.1 (±17.4)	

**Table 2:** Baseline characteristics of the restricted cohort.

	**PWS**		**Matched controls**	
	***N** =* **261**	**%**	***N** =* **13,050**	**%**
**Sex**				
Boys/Men	133	50.96%	6,650	50.96%
Girls/Women	128	49.04%	6,400	49.04%
**Cancer**				
Cancer at any age	5	1.92%	243	1.86%
Mean age at first cancer diagnosis (SD)	35.8 (±22.9)		38.7 (±16.7)	
Pediatric cancer (< 20 years)	1	0.38%	36	0.28%
**Emigration**				
Emigrated	5	1.92%	888	6.80%
Mean age at emigration (SD)	8.7 (±4.6)		15.1 (±12.1)	
**Death**				
Dead	19	7.28%	165	1.26%
Mean age at death (SD)	39.2 (±13.6)		20.6 (±18.3)	
31 December 2017	237	90.80%	12,000	91.95%
Mean age at the end of follow-up (SD)	21.7 (±14.0)		22.5 (±14.8)	

**Table 3:** Hazard ratios for the association between PWS and cancer at any age in the full and restricted cohorts.

**Full cohort**				**Restricted cohort**			
**Crude**		**Adjusted** ^*^		**Crude**		**Adjusted** ^*^	
**HR**	**95% CI**	**HR**	**95% CI**	**HR**	**95% CI**	**HR**	**95% CI**
1.09	0.61–1.93	1.07	0.60–1.89	0.98	0.40–2.37	0.95	0.39–2.31

^*^Birth year, sex, and parental educational level.

We observed a large proportion of germ cell tumors in young individuals with PWS. Besides the case presented below, a woman was diagnosed with an epithelial ovarian tumor at the age of 20 years and a young boy was diagnosed with a testicular embryonal carcinoma at the age of 17 years. The proportion of gonadal tumors (testicular or ovarian) among all individuals with cancer was 2/12 (17%) in PWS and 17/551 (3%) in the comparison group.

### 3.2. Case presentation

#### 3.2.1. Clinical report

A 13-year-old girl with PWS was referred to the genetics clinic with bilateral ovarian germ cell tumors. The patient did not have a family or personal history of cancer.

She and her twin sister were the first common children to non-consanguineous healthy parents. She has on her mother's and father's side, four and two healthy half-siblings, respectively. Pregnancy was uneventful, including a normal prenatal ultrasound examination and genetic screening for trisomy 13, 18, and 21. She was delivered at 37 weeks + 2 days of gestation by cesarean section due to breech presentation. Her birth weight was 2,510 g [−1.5 Standard Deviations (SD)], length 49 cm (+0.25 SD), and head circumference 33 cm (−0.75 SD). At birth, the patient was hypotonic and irritable, had weak reflexes and motor activity, and required continuous positive airway pressure support due to respiratory difficulties, as well as nasogastric tube feeding.

Upon physical examination, she was noted to have a distinct facial appearance with retro micrognathia, high palate, low-set ears, bitemporal narrowing, bilateral palmar creases, and slender hands and feet. Metabolic and infection investigations, ultrasound examination of the brain, heart, kidneys, and urinary tract, and an electroencephalogram were all normal. Genetic testing with chromosomal microarray and MS-MLPA revealed an imprinting defect due to a deletion of the paternal allele at the Prader-Willi region on chromosome 15 (15q11.2-q13). Her tonus and feeding difficulties gradually improved, and she was discharged home at 20 days of age.

Psychomotor development was mildly delayed. At 8 months of age, she was diagnosed with growth hormone deficiency and started replacement therapy, which continued until the age of 12 years. She received low-dose growth hormone treatment, initiating at 0.02 mg/kg/day and adjusting to 0.01–0.015 mg/kg/day after a year, due to her elevated insulin-like growth factor 1 (IGF-1) values, which went from about 90 μg/L before hormone replacement, to roughly between 275 and 570 μg/L during growth hormone treatment. The patient's parents also reported that she had difficulty sleeping and snored loudly at night. A sleep study revealed severe sleep apnea, and an adenotonsillectomy was performed with good results.

Between 1 and 2 years of age, the patient's body mass index rapidly increased from 19.2 kg/m^2^ (0 SD) to 21.6 kg/m^2^ (+3 SD), and she developed childhood hyperphagia (excessive appetite and obsession with eating). She was placed on a diet with a strict calorie limit to control her weight and prevent obesity. At 5 years of age, she developed hyperinsulinemia, her obsession with eating became more problematic, and episodes of rage were noted. At 6 years of age, she was diagnosed with intellectual disability and autism spectrum disorder.

The patient sought emergency care at 13 years of age for fever and abdominal pain. The parents also reported on a few-week-old history of hirsutism, hoarse voice, acne, and an episode of vaginal bleeding, which was interpreted as menarche. Laboratory investigations showed elevated C-reactive protein (282 mg/L), leukocytosis (14.8 × 10^9^/L) with neutrophilia (11.6 × 10^9^/L), anemia (Hb 100 g/L), and thrombocytosis (404 × 10^9^/L). An abdominal computed tomography scan revealed a right ovarian mass measuring 13 × 7.5 × 12.5 cm of heterogeneous aspect. Beta-hCG was elevated in serum (42 E/L), and alpha-fetoprotein was normal (>1 ug/L).

Abdominal magnetic resonance imaging confirmed the presence of a right ovarian tumor. X-ray and computed tomography scan of the thorax showed no lung metastasis. She was put on neoadjuvant chemotherapy and received a first cycle with bleomycin, etoposide, and cisplatin and a second cycle with etoposide, carboplatin, and bleomycin, after which the tumor could be surgically removed. Postoperatively, she received an additional cycle of chemotherapy. Six months after the surgery, a follow-up examination of the abdomen showed no evidence of tumor material or metastatic disease in the lymph nodes, and serum beta-hCG normalized. Clinical symptoms of virilization regressed.

The patient's height at 14 years of age was measured at 156.5 cm, which was 1 SD below the mean for her age and adequate according to her target height. Her weight was 79 kg, which was 3 SD above the mean for her age.

#### 3.2.2. Germline genetic analysis

At cancer diagnosis, germline WGS was performed on peripheral blood DNA to rule out potential childhood cancer predisposition syndromes. WGS confirmed the disease-causing deletion within the Prader-Willi critical region on 15q11.2-q13 (data not shown), but no additional pathogenic/likely pathogenic variants explaining the presence of multiple bilateral ovarian tumors in the patient were found.

#### 3.2.3. Histopathological and immunohistochemical assessment of the tumors

**Figure 1:**
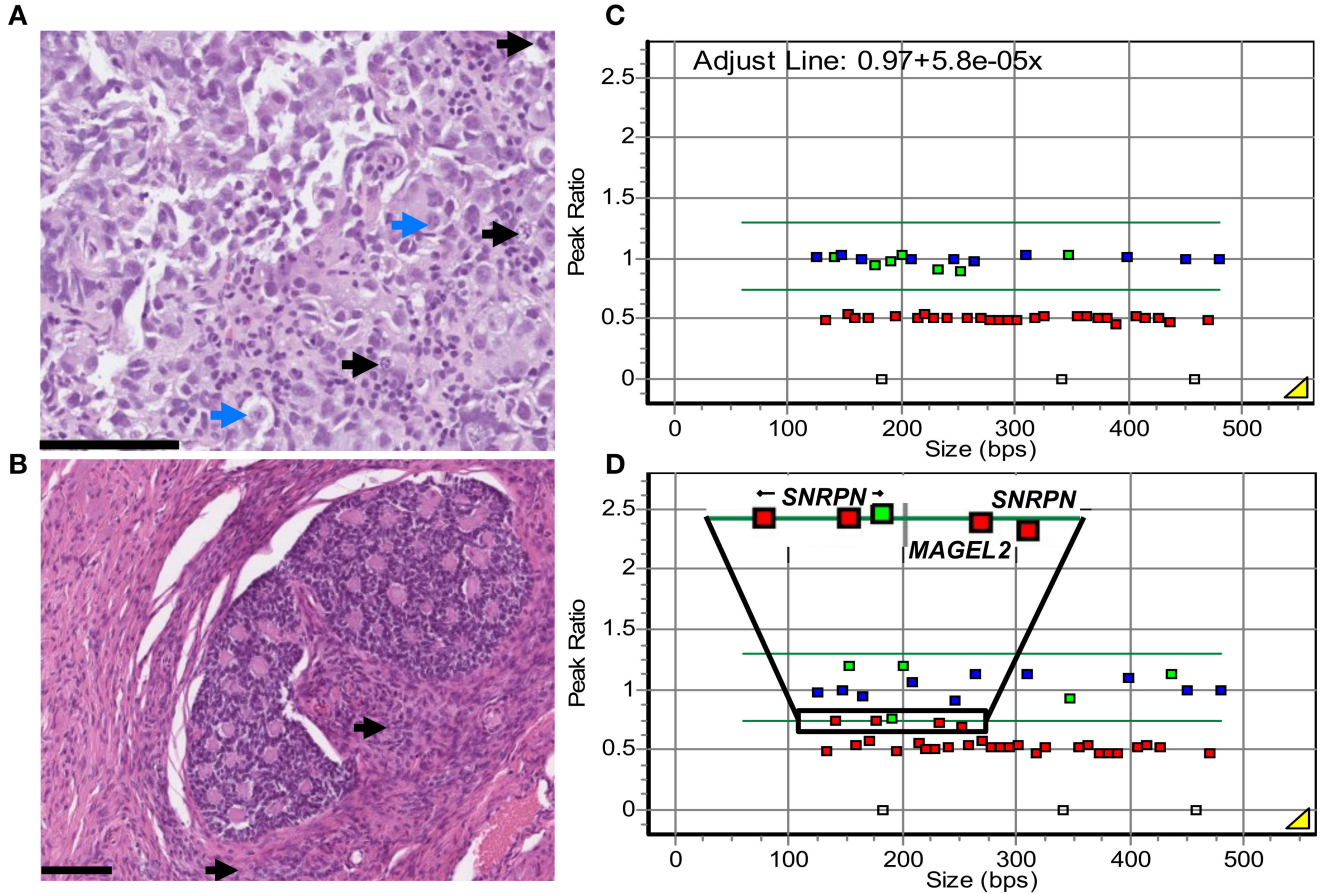
Patient's histological and genetic findings. **(A)** Diagnostic core biopsy from the right ovarian dysgerminoma, hematoxylin–eosin staining. Solid growing nests of atypical, monomorphic tumor cells with prominent nucleoli (blue arrows) and a moderate amount of clear to eosinophilic cytoplasm. Numerous mitoses were observed (black arrows). In the background, scattered tumor-infiltrating lymphocytes. Scale bar: 100 μm. **(B)** Surgical resection of right ovarian SCTAT visualized with hematoxylin-eosin staining. Nested formations of annular tubules with central eosinophilic, hyaline material. Focal Leydig cells (black arrows) and calcifications (not shown) were observed. Similar proliferations were found in the resected contralateral ovary. **(C, D)** MS-MLPA analysis of the PWS imprinted region 15q in DNA from the patient's blood **(C)** and the dysgerminoma **(D)**, after HhaI digestion. Control probes outside the 15q11 region are depicted in blue, target probes with peak ratio below 0.75 are depicted in red, and target probes with a peak ratio above 0.75 are depicted in green. Copy number probes confirm 15q11.2-q13 deletion (red squares, peak ratio 0.5 ± 0.25). In blood, the eight probes with HhaI show the expected methylation pattern, with a ratio of 1.0 ± 0.25 at the imprinted genes at 15q11.2-q13 (green squares). In the dysgerminoma, loss of methylation (peak ratio < 0.75) can be observed at four of these probes, at imprinted genes *SNRPN* and *MAGEL2* (selected region, red squares). A fifth probe presents a borderline value of 0.757 (selected region, green square).

Diagnostic fine needle aspiration biopsy from the right-sided ovarian tumor showed uniform medium to large-sized cells with atypical nuclei, prominent nucleoli, and finely vacuolated cytoplasm. Scattered lymphocytes were found in the background. Tumor cells were positive for OCT3/4, SALL4, and CD117 and negative for CD30 and Glypican 3, leading to a dysgerminoma diagnosis (data not shown). This was confirmed by the evaluation of core biopsies, performed simultaneously, which showed the solid growth of nested atypical monomorphic cells with nucleoli-containing nuclei and clear to eosinophilic cytoplasm. Focal necrosis and lymphocytes were also observed in the background (Figure 1A). Tumor cells were positive for OCT3/4, SALL4, and CD117 (SALL4 and CD117 staining in Supplementary Figures 1A, B) and negative for CD30 (data not shown).

After preoperative chemotherapy, bilateral oophorectomy was performed. The dysgerminoma in the right ovary was completely necrotic. In the adjacent ovarian stroma and contralateral ovary, there were well-circumscribed nests of cells with bland angulated nuclei and a moderate amount of cytoplasm with mild or no atypia or mitotic activity. Punched-out spaces containing eosinophilic material with focal calcifications were present (Figure 1B). These nested cells were variably positive for inhibin, calretinin, and SF1, while SALL4 was negative (inhibin and SFI staining in Supplementary Figures 1C, D). Sanger sequencing analysis for *FOXL2* C134W mutation was negative. In the presence of a dysgerminoma, gonadoblastoma was initially considered. However, this diagnosis was ruled out by the absence of germ cells on morphology and negative SALL4 staining. Instead, these sex cord proliferations were diagnosed as bilateral SCTATs.

#### 3.2.4. Genetic findings in the tumor tissue (dysgerminoma)

WGS profiling of the patient's ovarian dysgerminoma was performed to evaluate driver somatic genomic alterations, including copy number variations. The tumor presented a very complex copy number profile, with numerical and segmental aberrations in most of the chromosomes, except for chromosomes 5, 9, 11, 16, 18, and X. Multiple copies of chromosome 21 were observed. Chromosomes 1, 3, and 6 had clear breakpoints (Supplementary Figure 2). Finally, variant calling in the dysgerminoma detected a somatic pathogenic activating *KIT* mutation (NM_000222.3:c.1676T>G) with a variant allele frequency of 20% (Supplementary Figure 3A). No additional single-nucleotide variants were found in known cancer-related genes.

#### 3.2.5. Locus-specific loss-of-imprinting in the dysgerminoma

We investigated the imprinting status at the PWS region in the dysgerminoma by MS-MLPA. The results showed a normal ratio of five methylation-specific probes regarding the analysis of genomic DNA from blood in the PWS region (mean ratio 0.94, Figure 1C), as well as the imprinting region on chromosome 11 in blood and DNA from the dysgerminoma (Supplementary Figure 3B). Imprinting relaxation was implied in the tumor at the PWS region, where these five imprinted probes showed a ratio mean of 0.71, just below the normal threshold, but with a large difference compared to the analysis in blood (Figures 1C, D).

## 4. Discussion

Although PWS is not considered a cancer predisposition syndrome, there is preliminary evidence of an increased incidence of leukemia (16) and other malignancies (14, 15) in these patients. It has been debated whether growth hormone replacement treatment, leading to increased levels of IGF-1, may contribute to the observed increased cancer risk in PWS (31). In this study, we carried out a population-based cohort study investigating the frequency of cancer in individuals with PWS. We also report on an interesting clinical case of a 13-year-old girl with PWS due to a constitutional paternal deletion of the chromosomal region 15q11.2-q13, who developed both an ovarian dysgerminoma and bilateral SCTATs. Tumor identity was confirmed, while genetic analyses detected a somatic pathogenic activating *KIT* mutation and LOI at the PWS locus in the dysgerminoma.

In the population-based cohort study, the overall frequency of cancer was similar in PWS and age-matched comparisons, and time-to-event analysis did not indicate an overall increased risk of cancer in association with PWS. However, when considering the age at cancer onset, we found that the frequency of childhood cancer was three times higher in PWS than in age-matched controls (0.83 and 0.27%, respectively, in the full cohort). The numbers were too small to perform proper tumor subtype analyses, but when reviewing the specific cancer types, we found a large proportion of gonadal tumors among young individuals with PWS. Even if the increased childhood cancer incidence is below the risk level recommended for surveillance in the European Union (32), it is important that treating physicians are aware of this increased risk, especially since individuals with PWS may have difficulties expressing their health status.

Through a literature review of published reports of malignancies in individuals with PWS, we found a total of 50 described patients, including 13 individuals reported in the present study. Of note, only articles published before January 2022 were included in the review. Interestingly, the patients presented a considerably young age at cancer diagnosis, with an average of 24.5 years. Specifically, of the 46 reported cases with specified age at cancer diagnosis, 20 patients (43.5%) were diagnosed before the age of 20 years (Supplementary Table 1). This can result from a lower life expectancy in individuals with PWS (1, 33) or, as observed in the present study, an overrepresentation of pediatric malignancies in this patient population. As life expectancy increases in PWS, there is a possibility for further investigation of cancer risk in this patient group (17).

Of the 49 cases where cancer type was reported, 11 patients (22.5%) developed germ cell tumors (Supplementary Table 1). This is in line with results from previous studies, which indicate an elevated risk for testicular cancer in PWS (14, 15). All previous reports of germ cell tumors in PWS refer to testicular presentations (34–36). In this respect, a link between testicular malignancies and PWS has been proposed (34), possibly related to the high incidence of cryptorchidism in PWS (37), as an undescended testis increases the risk for testicular malignancies in male patients (38). However, dysgerminomas in females are equivalent to testicular seminomas in males, possibly indicating gonadal dysgenesis, rather than cryptorchidism, as the underlying cause. Three ovarian tumors were found in our study: one germ cell tumor and, notably, two gonadal but not germ cell ovarian tumors. Expressly, an ovarian epithelial tumor was found in our register-based study in addition to the reported patient's bilateral SCTATs.

We also performed tumor and germline genetic analysis in a 13-year-old girl with PWS who developed bilateral SCTATs and a unilateral ovarian dysgerminoma, since the presence of multiple primary tumors in childhood is one of the criteria for recognition of cancer predisposition syndromes (39). Interestingly, MS-MLPA revealed locus-specific LOI in the dysgerminoma at the PWS region, but not in a control region at 11p. This same phenomenon was previously reported in a child with PWS due to maternal UPD 15, who developed a germ cell testicular seminoma. He presented incomplete methylation at the PWS locus in the tumor, but not in iPSCs or skin fibroblasts (36). Similar to our study, the authors used the methylation status of a second imprinting region, i.e., the H19 cluster, to conclude that imprinting relaxation was locus-specific and possibly linked to carcinogenesis (36).

Imprinting relaxation has been previously described in cancer. Initial studies reported LOI in Wilms tumor at the imprinted region 11p (40, 41). Later on, imprinting relaxation in other cancer types was also documented (42), including in germ cell tumors (43). It is hypothesized that imprinting can result in cancer when the region that loses its epigenetic marks provides growing advantages to the cell or when the only expressed copy of a TSG losses its function (13). At 11p, LOI results in overexpression of the growth factor *IGF2*, leading to increased cell division and growth (44). A similar mechanism could possibly explain the link between LOI at the imprinted region 15q11.2-q13 and tumorigenesis.

We queried the coding genes located in the PWS imprinted region [*MKRN3, MAGEL2, NDN, NPAP1*, and *SNURF-SNRPN* (1)] in public databases of cancer driver genes. As of June 2023, none of the genes was found in the Census Tiers from the COSMIC database (45), while three were candidate cancer drivers according to the Network of Cancer Genes (46). Of these, *NPAP1* and *NKRN3* are putative TSG, with prevalent loss-of-function alterations in melanoma and lung cancer, respectively. The third gene, *SNRPN*, is considered a putative oncogene, due to prevalent gain-of-function alterations and duplications in cancer. *In vitro* studies in cancer cell lines showed that increased cell proliferation, metastatic capability, and cell cycle progression positively associate with *SNRPN* expression (47–49). Finally, the *NDN* gene is reported by the Tumor Suppressor Gene Database as a potential TSG (50). Necdin, the protein encoded by the *NDN* gene, arrests cell cycle progression and interacts with TP53 to inhibit cell growth (51). It has been hypothesized that the lack of NDN in patients with PWS may lead to cancer predisposition (17). We thus conclude that carcinogenesis in patients with PWS could be linked either to *SNRPN* re-expression or changes in *NDN* expression in patients with PWS. Moreover, the involvement of additional genetic factors or growth hormone replacement treatment (31) in cancer development cannot be excluded. Therefore, follow-up functional studies are needed to understand the impact of LOI at 15q11.2-q13 in carcinogenesis.

Germline WGS analysis in our patient did not lead to the identification of additional disease-causing variants besides the 15q11.2-q13 paternal deletion. However, a pathogenic missense *KIT* activating variant, previously not described in germ cell tumors, was discovered in the dysgerminoma of the 13-year-old girl. *KIT* encodes a tyrosine kinase receptor involved in cell differentiation and germ cell survival. In cancer, it has been associated with the presence of cancer stem cells and increased epithelial-mesenchymal transition (52). Furthermore, *KIT* amplification or somatic activation (due to mutations in exon 17, codon 816) is present in 27–53% of ovarian dysgerminomas (53–55). Furthermore, *KIT* pathogenic variants associated with tumor development are found in 10–40% of testicular seminomas (55). Based on the present results, it is not possible to determine whether the initial driving event in the dysgerminoma corresponds to the somatic *KIT* variant or LOI at 15q.

As in the previously published epidemiological investigations of cancer in association with PWS, the main limitation of the present study is the cohort size. Although the Swedish registry holds records of citizens from 1960s onwards, only individuals diagnosed with PWS between 1997 and 2017 are included in the study, since only ICD-10 diagnosis of PWS is reliable. Additionally, both PWS and pediatric cancer are rare diseases, thus reducing the number of reported cases to < 5. A similar limitation was encountered when analyzing the incidence of germ cell malignancies in PWS. Furthermore, as adult cancer correlates with increasing age, the relatively short follow-up time may have contributed to the underestimation of the true association between PWS and cancer in adulthood. Therefore, future studies on larger cohorts will be needed to validate the pediatric and lifetime cancer risks in individuals with PWS.

In conclusion, we found a high frequency of pediatric cancer, especially gonadal tumors, in individuals with PWS, suggesting an increased risk for these malignancies in PWS. Studies on larger cohorts with longer follow-up times are needed to clarify the lifetime cancer risk in this patient group. Furthermore, we have presented the second case of locus-specific imprinting relaxation in a germ cell tumor in a patient with PWS and suggest LOI as a possible mechanism for tumorigenesis in these patients.

## Data availability statement

The data supporting the findings of this study are available as Supplementary material or on request from the corresponding authors, CM-G or AN. Some data are not publicly available as it contains information that could compromise the privacy of the research participants.

## Ethics statement

The studies involving human participants were reviewed and approved by the Regional Ethical Review Board in Stockholm (Dnr 2015/292-31/4, Dnr 2018/1849-32, Dnr 2019-04746, and Dnr 2021-05916-02). Written informed consent to participate in this study was provided by the participants' legal guardian/next of kin.

## Author contributions

AN, AS, and EK contributed to the conception and design of the study. SH, RN, AD-V, and CH contributed to data acquisition. AS and GT performed the statistical analysis. KL-R, FT, BT, AP, AN, and CM-G performed germline WGS/MLPA analyses. SW, TD, KL-R, FT, CM-G, and AN performed somatic WGS/MLPA analyses. SW and WM performed pathological examinations. CM-G, AS, and AN wrote the first draft of the manuscript. AS, KL-R, CM-G, AD-V, and SW prepared figures and tables and wrote sections of the manuscript. SH and RN provided medical care for the patient. All authors contributed to the manuscript revision and read and approved the submitted version.
