# Supplementary material for: Register-based and genetic studies of Prader-Willi syndrome show a high frequency of gonadal tumors and a possible mechanism for tumorigenesis through imprinting relaxation
Source: Front Med (Lausanne). 2023 Jul 28;10:1172565. doi: 10.3389/fmed.2023.1172565 (PMC10419300; doi:10.3389/fmed.2023.1172565)
Supplement: Supplementary file 1 [file Data_Sheet_1.docx]

Supplementary Figures

**Register-based and genetic studies of Prader-Willi syndrome show a high frequency of gonadal tumors and a possible mechanism for tumorigenesis through imprinting relaxation**


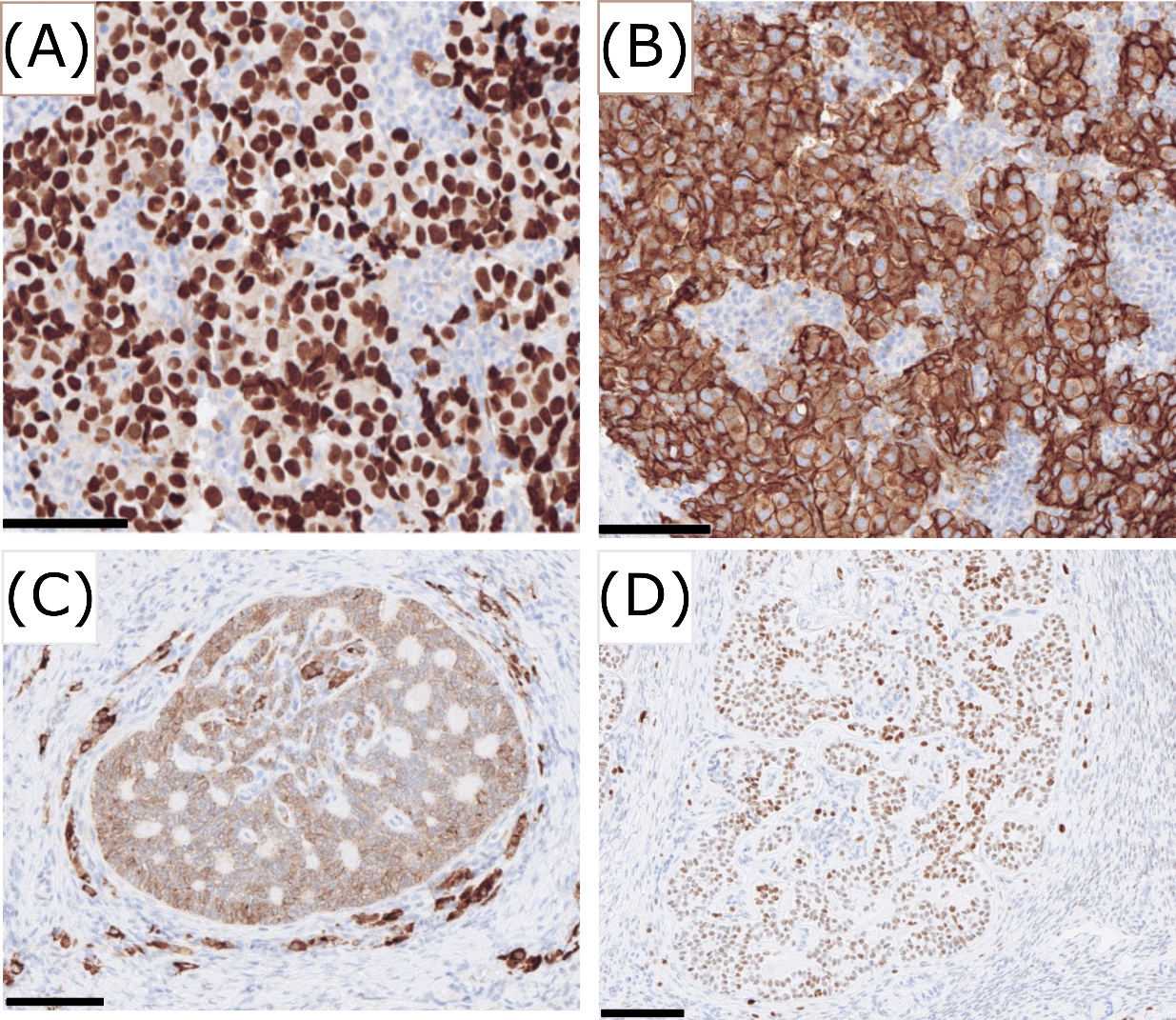


**Supplementary Figure 1. Immunohistochemical findings of the ovarian tumors. (A-B)** Dysgerminoma positive for SALL4 **(A)** and CD117 **(B)**. **(C)** Positive inhibin immunostaining in sex cord tumor with annular tubules (SCTAT) showing annular tubes in light brown surrounded Leydig cells stained in dark brown. **(D)** SFI positive immunostaining in SCTAT. Scale bars: 100 µm. Contralateral SCTAT presented a similar pattern (Not shown).


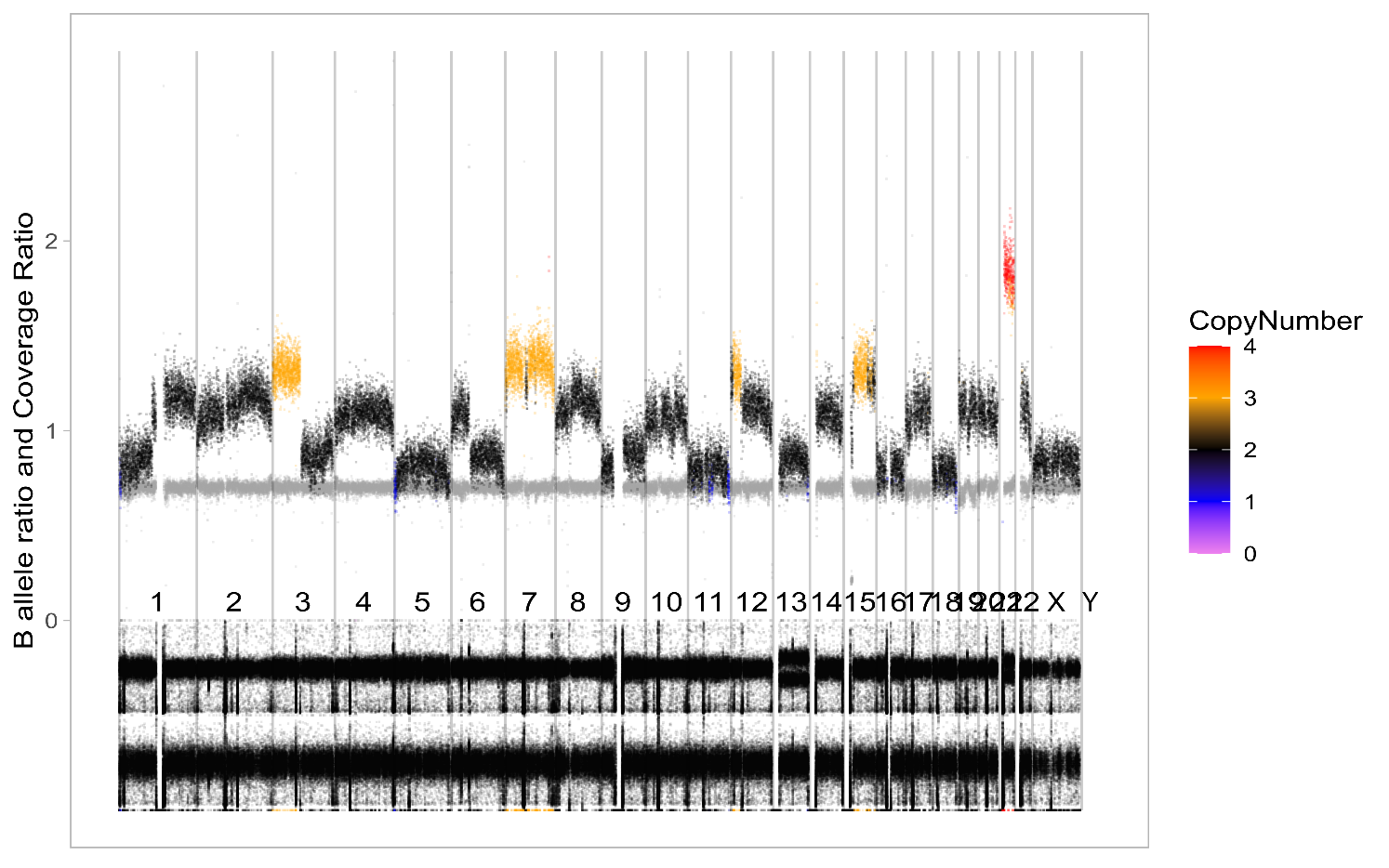


**Supplementary Figure 2.** **Copy number changes in the dysgerminoma.** Overlaid normalized coverage ratio profiles for the ovarian dysgerminoma (Black track) and matched blood sample (Grey track), as determined by Control Freec v11.6 from Whole Genome Sequencing (WGS) data. Below, the B allele ratio for heterozygous SNVs in tumor and blood samples is presented for each chromosome. The color key for the predicted B allele ratios is presented to the right of the figure. Regions with allelic balance present a ratio of 0.5 and areas of allelic imbalance (as chromosomes 13 and 21) present intermediate values. Numerical and segmental aberrations can be detected in most chromosomes, except chromosomes 5, 9, 11, 16, 18 and X. Multiple copies of chromosome 21 can be observed. Chromosomes 1, 3, and 6 show clear breakpoints.


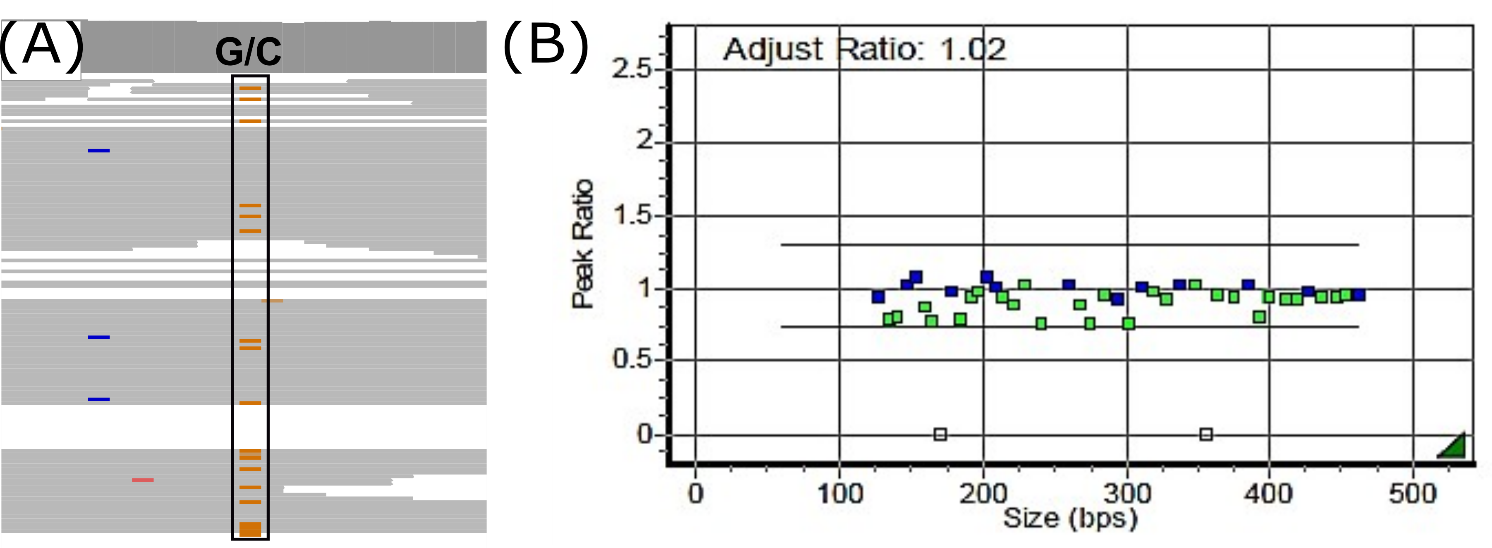


**Supplementary Figure 3**. **Additional genetic and methylation findings in the dysgerminoma.** **(A)** IGV pileup at the site of the somatic pathogenic activating *KIT* mutation (NM_000222.3:c.1676T>G) in the ovarian dysgerminoma. The variant presents an allele frequency of 20% and was not detected in germline DNA (Not shown). **(B)** MS-MLPA results at the Beckwith-Wiedemann /Silver Russel Syndrome imprinted region in chromosome 11p in the dysgerminoma, after HhaI digestion. Results show a normal methylation pattern, with a ratio of 1.0±0.25.
